# Impact of Probiotics on the Salivary Microbiota and Salivary Levels of Inflammation-Related Proteins during Short-Term Sugar Stress: A Randomized Controlled Trial

**DOI:** 10.3390/pathogens10040392

**Published:** 2021-03-25

**Authors:** Christine Lundtorp-Olsen, Christian Enevold, Claus Antonio Juel Jensen, Steen Nymann Stofberg, Svante Twetman, Daniel Belstrøm

**Affiliations:** 1 Department of Odontology, Section for Clinical Oral Microbiology, Faculty of Health and Medical Sciences, University of Copenhagen, 2200 Copenhagen, Denmark; christine-lundtorp@hotmail.com (C.L.-O.); stwe@sund.ku.dk (S.T.); 2 Center for Rheumatology and Spine Diseases, Institute for Inflammation Research, Rigshospitalet, Copenhagen University Hospital, 2100 Copenhagen, Denmark; chrej@yahoo.dk; 3 Department of Clinical Biochemistry, Nordsjællands Hospital, 3400 Hillerød, Denmark; claus.antonio.juel.jensen@regionh.dk (C.A.J.J.); steen.nymann.stofberg@regionh.dk (S.N.S.)

**Keywords:** oral microbiota, sugar stress, homeostasis, 16S rDNA

## Abstract

Background: The purpose of the present investigation was to characterize the effect of probiotics on the composition of the salivary microbiota and salivary levels of inflammation-related proteins during short-term sugar stress. We tested the hypotheses that consumption of probiotics may partly counteract the detrimental influence of sugar stress on oral homeostasis. Methods: The present study was a five-week, blinded, randomized controlled trial with four study arms—A: sucrose and probiotic (*n* = 20); B: sucrose and placebo (*n* = 20); C: xylitol and probiotic (*n* = 20); D: xylitol and placebo (*n* = 20). Saliva samples were collected at baseline and after two and five weeks. The salivary microbiota was characterized by means of 16S rDNA sequencing, and sequences were referenced against the Human Oral Microbiome Database (HOMD). Neutrophil gelatinase-associated lipocalin (NGAL) and transferrin levels were quantified using immunoassays. Results: Sugar stress induced a significant increase in the relative abundance of the genus *Streptococcus* from 29.8% at baseline to 42.9% after two weeks. Changes were transient and were completely reversed three weeks after discontinuation of sugar stress. Xylitol and probiotics alone had no effect on the salivary microbiota, whereas the combination of xylitol and probiotics induced a significant decrease in the relative abundance of *Streptococcus* species from 37.6% at baseline to 23.0% at week 2. Sugar stress significantly increased salivary transferrin levels, and the effect was partly counteracted by concomitant use of probiotics. Conclusions: The data clearly demonstrate an impact of combined consumption of xylitol and probiotics on the composition of the salivary microbiota. Future studies are needed to evaluate whether the combined use of xylitol and the probiotic strains tested could have clinically protective effects during periods of sugar stress.

## 1. Introduction

Oral health is determined by a symbiotic relationship between the oral microbiota and the immune system of the host [1,2]. However, internal and external perturbations, which affect either the microbiota or the host, will challenge oral homeostasis by alterations in ecological conditions, which might, in turn, induce structural alterations of the oral microbiota that may subsequently lead to development of oral diseases such as dental caries [1,2,3]. 

Dental caries is a biofilm-mediated multifactorial disease, where the key pathogenic acts are the complex interaction between the oral microbiota, the availability of free carbohydrates, saliva and genetic factors [4]. In particular, sucrose has been documented to play a crucial role in the initiation and progression of dental caries, as sucrose induces compositional changes of the supragingival plaque microbiota, favoring overgrowth of acidic and acidophilic species, which leads to loss of diversity of the biofilm [1,3,5]. In contrast, non-fermentable sweeteners and sugar-alcohols such as xylitol may have a preventive effect on dental caries development. For example, xylitol can stimulate saliva secretion, which may, therefore, augment the natural defense mechanisms of saliva. Likewise, as the end-products of xylitol fermentation are not acidic, intake of xylitol does not stimulate overgrowth of potential cariogenic members of the oral microbiota [6]. 

Probiotics are defined by World Health Organization (WHO) as “live microorganisms which when administered in adequate amounts, confer a health benefit on the host”. The scientific interest in probiotics for prevention of dental caries has emerged rapidly in recent years [7]. While the exact working mechanisms of probiotics remain unclear, these are categorized as direct and indirect. Indeed, from a cariogenic perspective, especially the direct, local effect is interesting, where several clinical trials have reported an increase in pH in saliva and a concomitant reduction in *Streptococcus mutans* levels [8,9,10,11]. On the other hand, the indirect systemic effect of probiotics is less investigated, but is believed to be conveyed by interactions with the host’s immune system [7]. 

The long-term detrimental clinical consequences of frequent carbohydrate consumption have been known since the highly controversial Vipeholm investigation was conducted in the 1950s [12]. However, less is known on the immediate impact of sugar stress on oral homeostasis, and adequately powered in vivo studies characterizing the effect of excessive carbohydrate intake on the composition of the oral microbiota in orally healthy individuals are missing. Indeed, saliva, which can be sampled with ease and non-invasively, portrays the composition of the oral microbiota and harbors relevant inflammatory markers [13,14]. Importantly, the composition of the salivary microbiota and salivary levels of inflammatory markers have been demonstrated not only to reflect oral health status [15,16], but also to be susceptible to external stress such as oral hygiene discontinuation, smoking and diet [17,18,19]. Therefore, analysis of the salivary microbiota and salivary levels of inflammatory markers is an elegant model system to study the impact of coordinated perturbations on oral homeostasis. However, to the best of our knowledge, this model system has not previously been used to characterize the short-term effect of frequent carbohydrate consumption on oral homeostasis and to disentangle if concomitant use of probiotics has any protective effect when oral homeostasis is stressed by frequent sugar intake. 

The purpose of the present investigation was to characterize the effect of probiotics during short-term sugar stress on oral homeostasis. We tested the hypothesis that concomitant consumption of two probiotic strains, *Lacticaseibacillus rhamnosus* (formerly *Lactobacillus*) PB01 DSM14870 and *Latilactobacillus curvatus* (formerly *Lactobacillus*) EB10 DSM32307, may partly counteract the detrimental influence of short-term sugar stress, as evaluated by compositional changes of the salivary microbiota and salivary levels of inflammation-related proteins. 

## 2. Results

### 2.1. Background Data

Background data of the study population are presented in Table 1. As can be seen, no difference in general parameters such as age and gender distribution was evident.

### 2.2. Sequencing Metadata

A total of 16 samples failed quality controls. Thus, a total of 41,936 reads per sample were included in downstream analysis, including 4115 unique operational taxonomic units (OTUs), which were identified from 9.2 million reads retrieved from a total of 219 samples. A total of 413 different bacterial species and 111 bacterial genera were identified, corresponding to 61.5% and 96.3% coverage of the generated sequences, respectively. A mean Alpha diversity, as determined by Shannon index, of 4.3 was observed. 

### 2.3. Short-Term Sugar Stress Changes the Composition of the Salivary Microbiota

**Figure 1:**
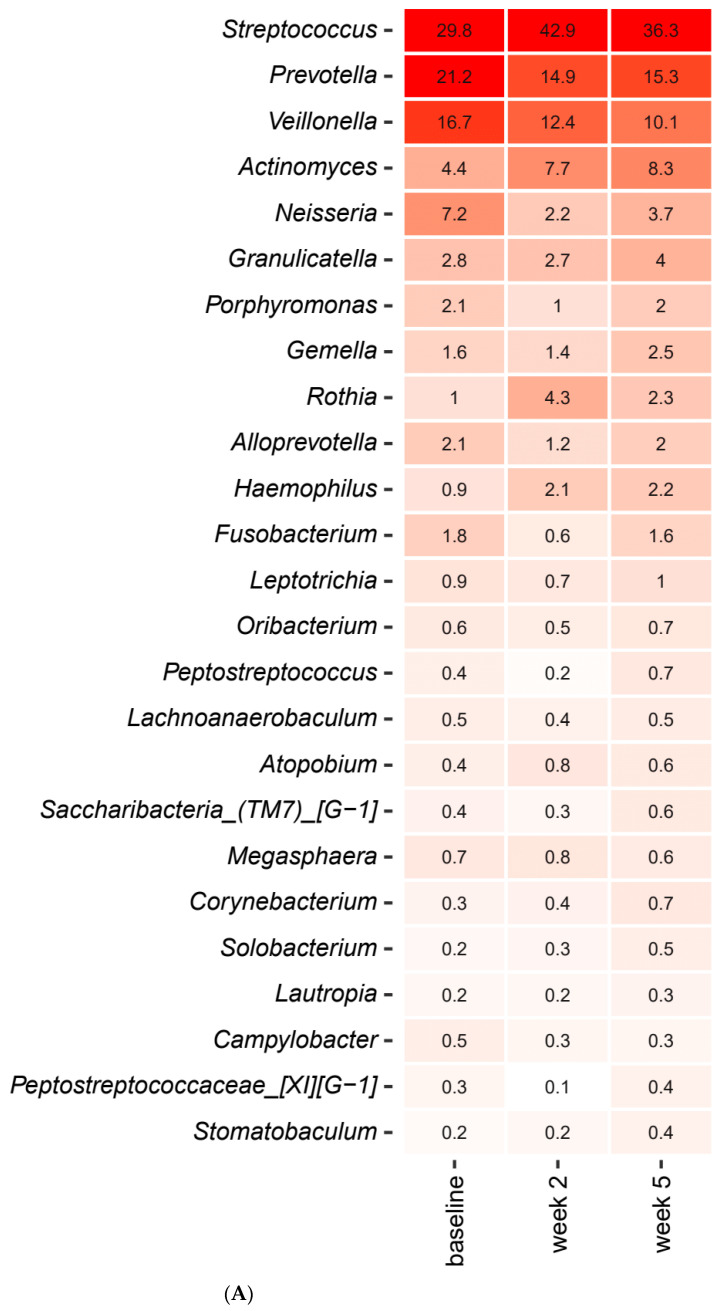

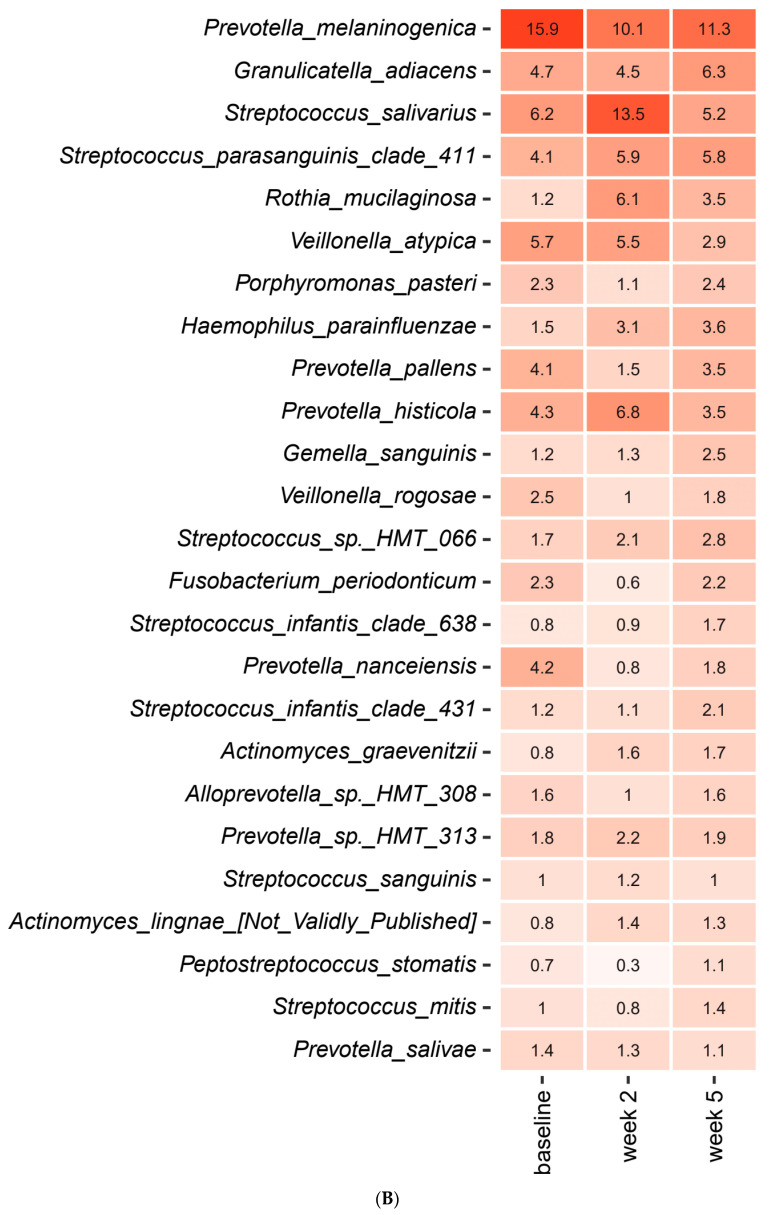

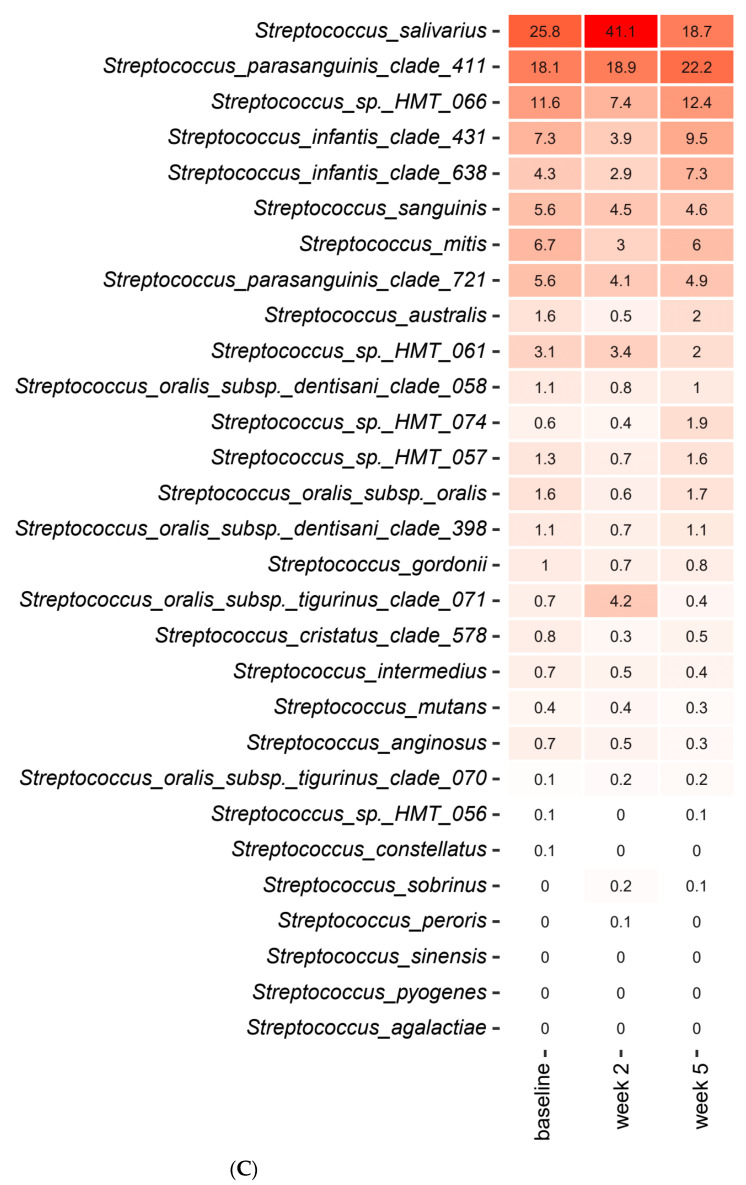
Impact of sugar stress on predominant microbiota. Mean values of relative abundance of top 25 predominant genera (**A**), species (**B**) and *Streptococcus* species (**C**) in the sucrose and placebo group recorded at baseline, week 2 and week 5. The intensity of the red color denotes the level of relative abundance.

Short-term sugar stress with and without concomitant use of probiotics had an immediate impact on the composition of the salivary microbiota. Figure 1A,B detail the relative abundance of the core salivary microbiota at baseline, after two weeks of sugar stress and three weeks after discontinuation of sugar stress (week 5), expressed as mean values of predominant genera (Figure 1A) and species (Figure 1B). As seen, short-term sugar stress had a major impact on the composition of the core salivary microbiota, with significant increases in relative abundance of *Streptococcus, Actinomyces* and *Rothia* species in combination with significant decreases in *Prevotella*, *Neisseria* and *Fusobacterium* species after two weeks sugar stress, which were all gradually revised three weeks after discontinuation of sugar stress (week 5). Figure 1C details the mean values of relative abundance of predominant *Streptococcus* species at each time point. As can be seen, short-term sugar stress resulted in a significant increase in the relative abundance of *Streptococcus salivarius* from 25.8% at baseline to 41.1% at week 2, which went back to baseline conditions when the sugar stress was removed (Figure 1C).

**Figure 2:**
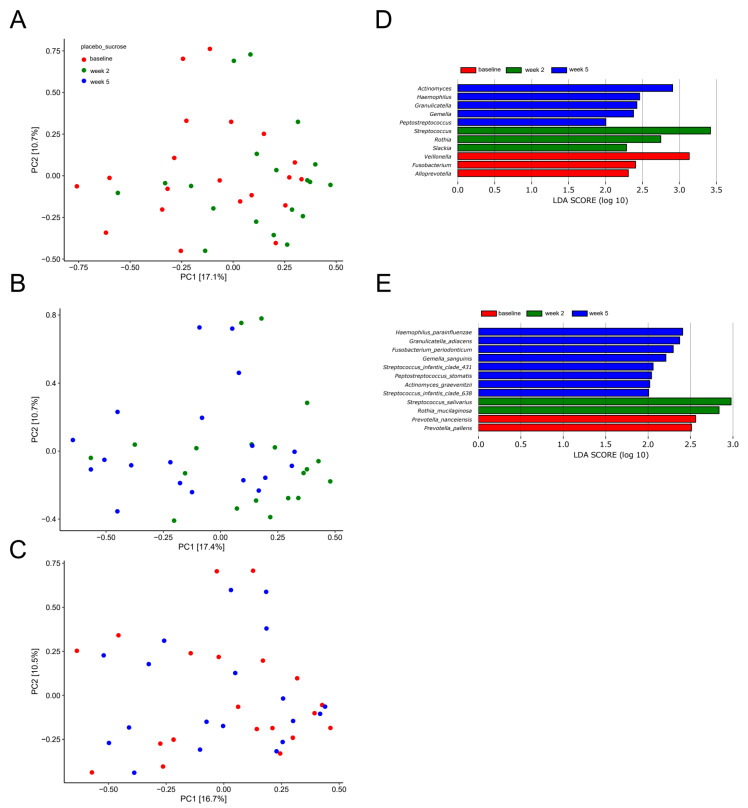
Compositional changes induced by sugar stress. Principal component analysis (PCA) expressed by the two most decisive variables (PC1 and PC2) accounting for approx. 27% of the variation of the dataset in the sucrose and placebo group. (**A**) baseline vs. week 2. (**B**) week 2 vs. week 5. (**C**) baseline vs. week 5. Linear discriminant analysis effect size (LEfSe) analysis expressed by significant genera (**D**) and species (**E**).

A principal component analysis (PCA) based on the two most decisive components (PC1 and PC2), which collectively covered approximately 27% of the variation of the dataset, showed almost complete separation of samples collected at baseline and after two weeks of sugar stress (Figure 2A). Likewise, separation of samples from week two and samples collected three weeks after discontinuation of sugar stress (week 5) was evident (Figure 2B). On the other hand, a completely random distribution of baseline samples and samples collected three weeks after discontinuation of sugar stress (week 5) was observed (Figure 2C). 

Figure 2D,E show bacterial genera and bacterial species which, by means of linear discriminant analysis effect size (LEfSe) analysis, were identified to be significantly impacted by sugar stress. As can be seen, short-term sugar stress favored *Streptococcus salivarius* and *Rothia mucilaginosa*, which were then outnumbered by *Haemophilus* and *Actinomyces* species when the sugar stress was removed.

Simultaneous use of probiotics had no protective effect when the salivary microbiota was stressed by frequent carbohydrate intake. Accordingly, the abovementioned differences were also evident in the group which received probiotics during the period of sugar stress (data not shown). 

### 2.4. Xylitol Has Minimum Effect on the Composition of the Salivary Microbiota

**Figure 3:**
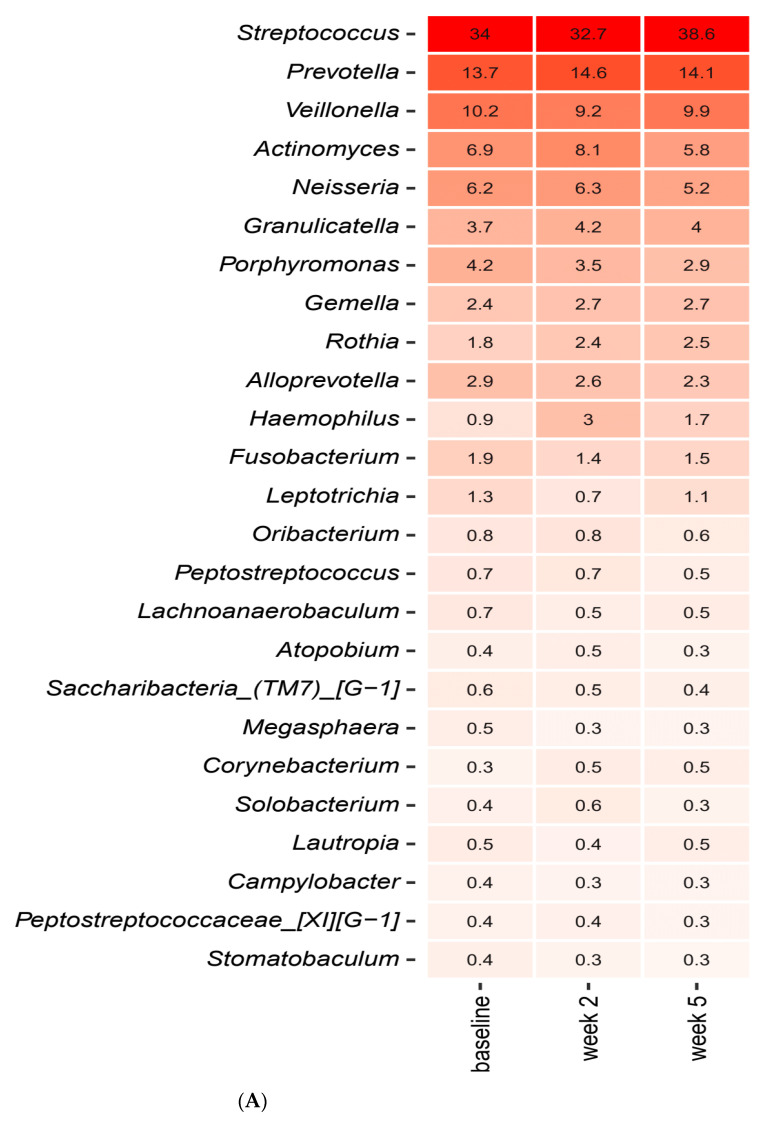

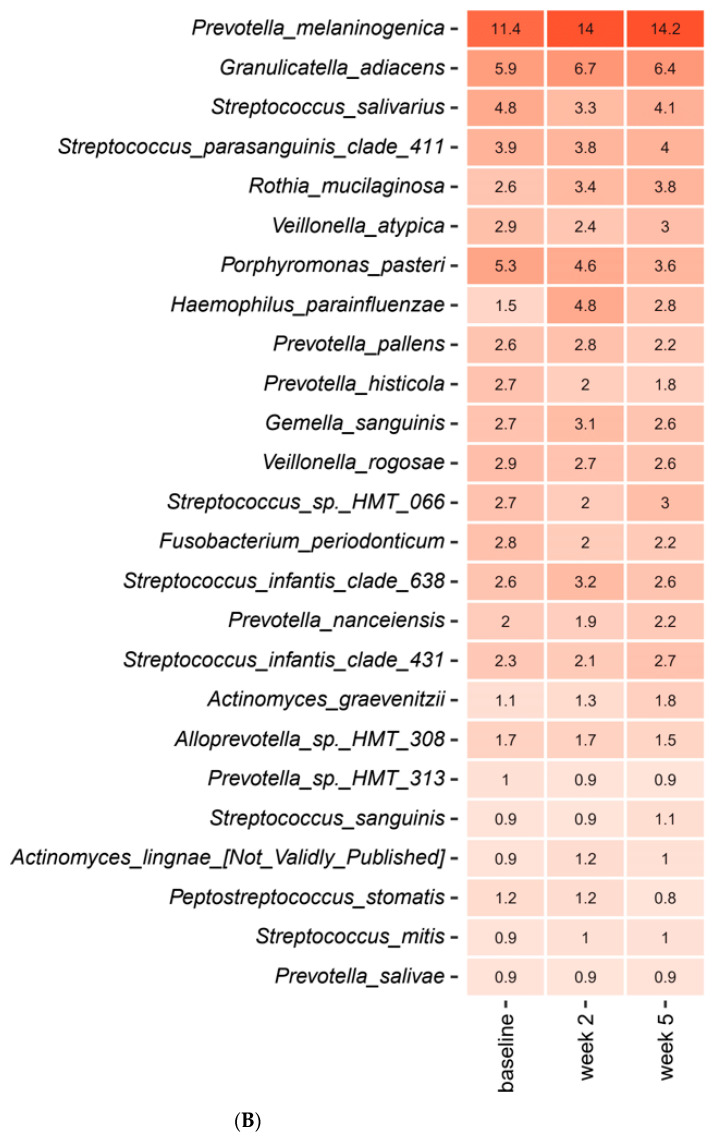

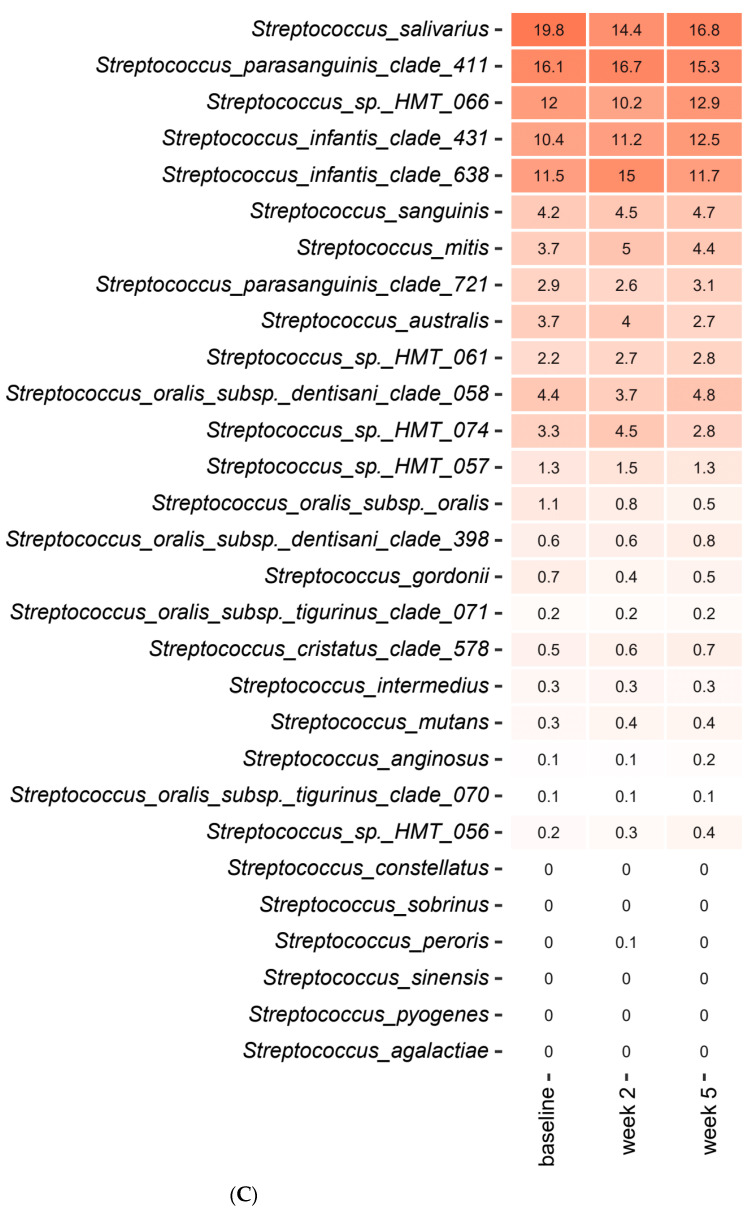
Impact of xylitol on predominant microbiota. Mean values of relative abundance of top 25 predominant genera (**A**), species (**B**) and *Streptococcus* species (**C**) in the xylitol and placebo group recorded at baseline, week 2 and week 5. The intensity of the red color denotes the level of relative abundance.

Figure 3A,B detail the relative abundance of the core salivary microbiota before and after a two-week xylitol stress, visualized as mean values of predominant genera (Figure 3A) and species (Figure 3B). As seen, short-term xylitol stress had no impact on the composition of the core salivary microbiota. Likewise, no impact of xylitol on the composition of predominant *Streptococcus* species was observed (Figure 3C). 

**Figure 4:**
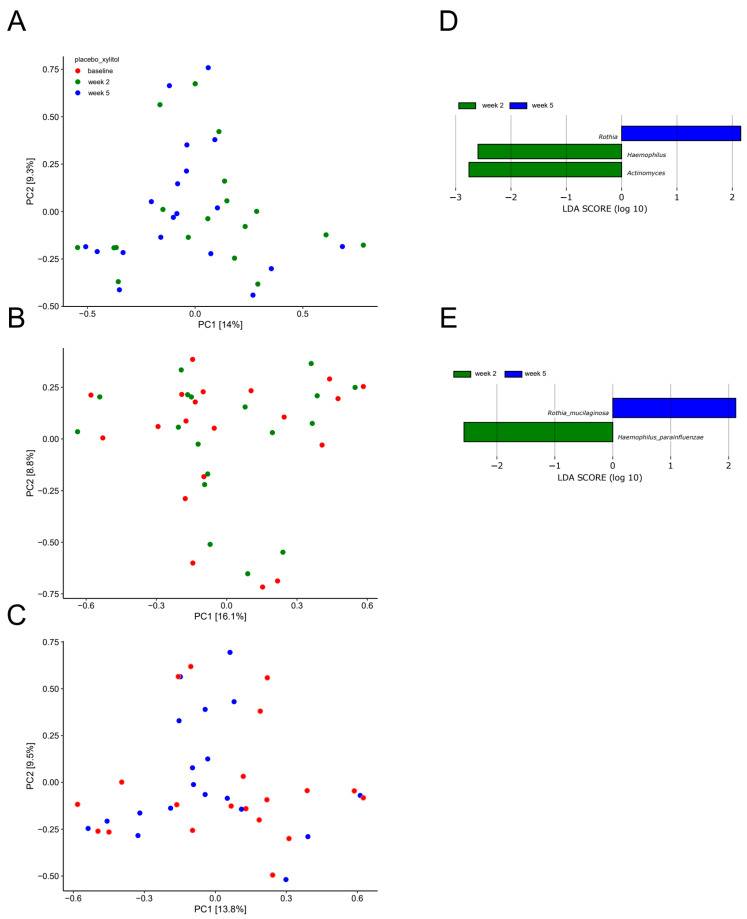
Compositional stability during xylitol stress. PCA expressed by the two most decisive variables (PC1 and PC2) accounting for approx. 23% of the variation of the dataset in the xylitol and placebo group. (**A**) baseline vs. week 2. (**B**) week 2 vs. week 5. (**C**) baseline vs. week 5. LEfSe analysis expressed by significant genera (**D**) and species (**E**).

In addition, random distribution of samples as determined by PCA (Figure 4A−C), in combination with identification of only two significant bacterial species (*Haemophilus parainfluenzae* and *Rothia mucilaginosa)* as evaluated by LEfSe (Figure 4D–E), confirmed the minimal impact of xylitol on the salivary microbiota.

### 2.5. Probiotics in Combination with Xylitol Have a Major Impact on the Composition of the Salivary Microbiota 

**Figure 5:**
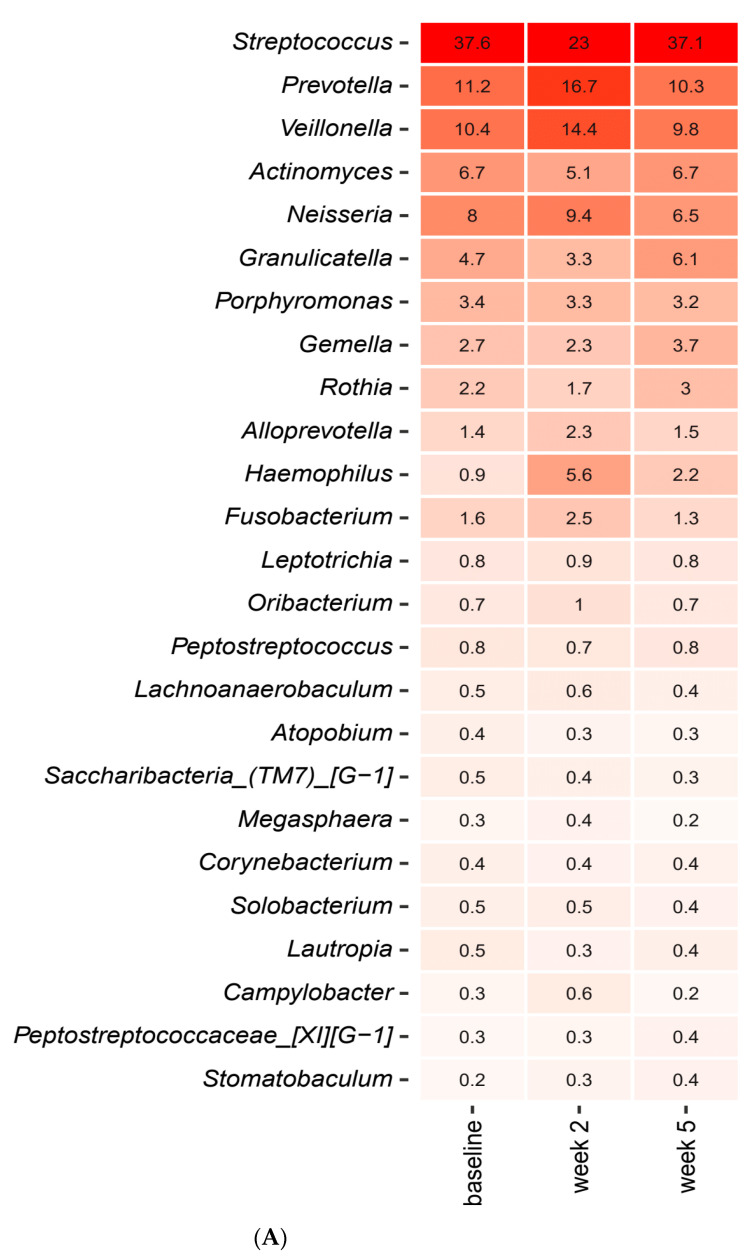

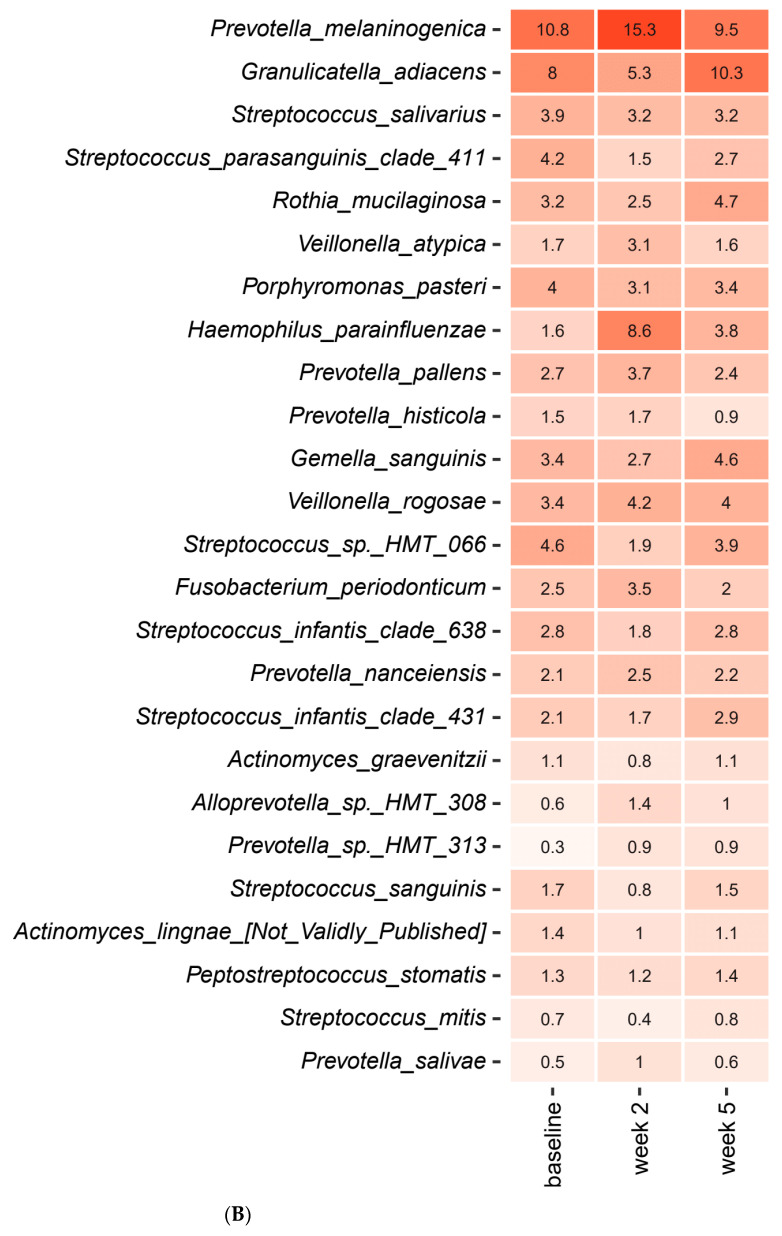

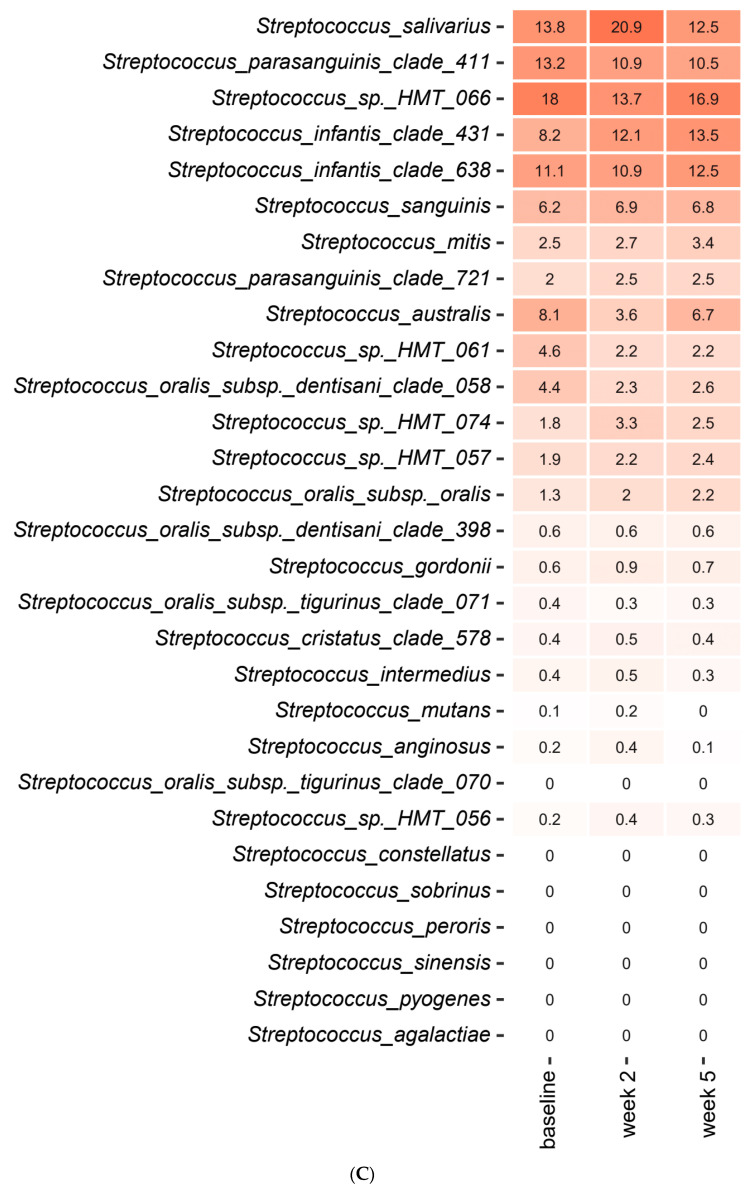
Impact of xylitol and probiotics on predominant microbiota. Mean values of relative abundance of top 25 predominant genera (**A**), species (**B**) and *Streptococcus* species (**C**) in the xylitol and probiotics group recorded at baseline, week 2 and week 5. The intensity of the red color denotes the level of relative abundance.

Figure 5A,B detail the relative abundance of the core salivary microbiota in the probiotics group at baseline, after two weeks of combined use of probiotics and xylitol and after three weeks usage of probiotics alone (week 5), expressed as mean values of predominant genera (Figure 5A) and species (Figure 5B). As can be seen, the combination of probiotics and xylitol had a major impact on the core salivary microbiota as visualized by a significant decrease in *Streptococcus* species and a concomitant significant increase in *Prevotella*, *Haemophilus* and *Fusobacterium* species, which was then completely reversed when xylitol was discontinued (week 5). Figure 5C details the relative abundance of predominant *Streptococcus* species at each time point. As can be seen, the combination of probiotics and xylitol resulted in a significant increase in the relative abundance of *Streptococcus salivarius,* whereas other *Streptococcus* species such as *australis* and *oralis* decreased. Nevertheless, all changes were completely reversed three weeks after xylitol had been discontinued (week 5).

**Figure 6:**
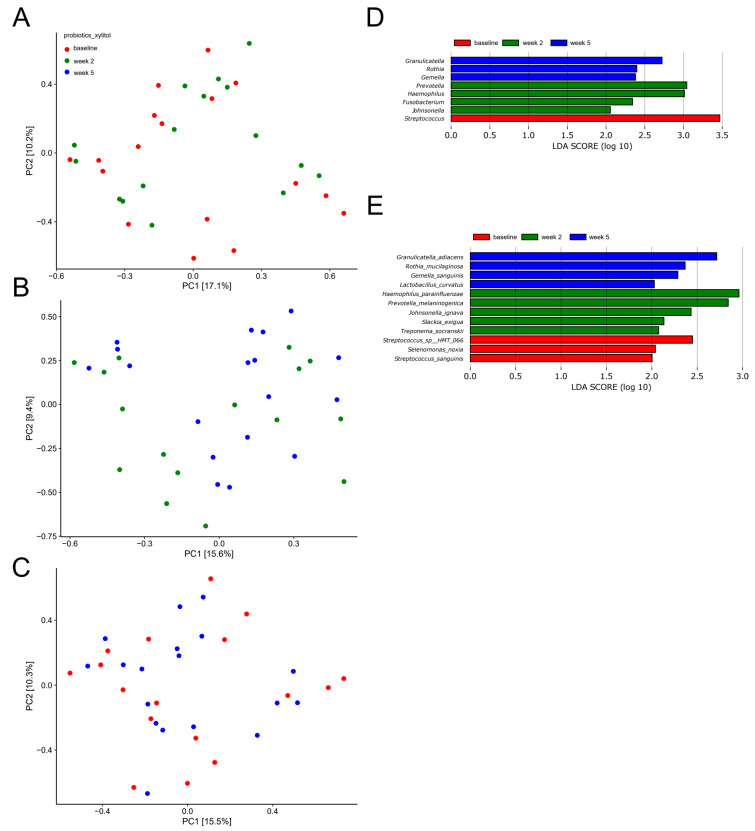
Compositional changes induced by xylitol and probiotics. PCA expressed by the two most decisive variables (PC1 and PC2) accounting for approx. 27% of the variation of the dataset in the xylitol and probiotics group. (**A**) baseline vs. week 2. (**B**) week 2 vs. week 5. (**C**) baseline vs. week 5. LEfSe analysis expressed by significant genera (**D**) and species (**E**).

PCA showed fair separation of baseline samples and samples collected after two weeks of combined use of probiotics and xylitol (Figure 6A). Likewise, week 2 samples and samples collected at three weeks after discontinuation of xylitol (week 5) showed some degree of separation (Figure 6B). On the other hand, a completely random distribution of baseline samples and samples collected three weeks after discontinuation of xylitol (week 5) was observed (Figure 6C). 

Figure 6D,E display the bacterial genera and bacterial species which were significantly altered as evaluated by LEfSe analysis. As can be seen, the combination of probiotics and xylitol favored *Prevotella, Haemophilus* and *Fusobacterium* species, which were then followed by *Granilucatella, Rothia* and *Gemella* species when xylitol was discontinued.

### 2.6. Impact of Sucrose, Xylitol and Probiotics on Salivary Levels of NGAL and Transferrin

Median levels and 95% CI of neutrophil gelatinase-associated lipocalin (NGAL) and transferrin (ng/mL) at each time point in the four groups are presented in Table 2 and Table 3, respectively. As can be seen, none of the perturbations had any significant impact on salivary levels of NGAL (Table 2). On the other hand, sucrose resulted in a significant increase in median salivary levels of transferrin from baseline (4.5) to week 2 (5.5), which continued to increase to week 5 (6.8), even after the sucrose perturbation had been stopped. On the contrary, sucrose had no effect on median salivary levels of transferrin when probiotics were also consumed. In addition, xylitol showed a borderline significant lowering effect on median salivary levels of transferrin when administrated in combination with probiotics (Table 3). 

## 3. Discussion

The purpose of the present randomized controlled trial was to characterize the impact of probiotics on the salivary microbiota and salivary levels of inflammation-related proteins during short-term sugar stress. The main finding was that the combined consumption of xylitol and the selected probiotic strains induced a significant decrease in relative abundance of *Streptococcus* species, which was opposite to the changes to the salivary microbiota mediated by short-term sugar stress. Consequently, combined use of xylitol and probiotics may offer a potential clinically relevant adjunct to conventional caries prevention methods in individuals with frequent carbohydrate consumption. 

Sucrose rinsing was employed to stress the oral homeostasis. The perturbation of frequent sucrose rinsing had an immediate impact on the composition of the salivary microbiota, as was clearly illuminated by PCA, which demonstrated separation of the samples collected after two weeks of sugar stress as compared to the corresponding baseline samples (Figure 2A). Indeed, this finding was not surprising, since sugar intake is well known to favor bacterial proliferation in the oral cavity [20]. However, to the best of our knowledge, this is the first large-scale clinical trial demonstrating the impact of sugar intake on the salivary microbiota. Specifically, sugar stress induced a significant increase in relative abundance of the genus *Streptococcus* from 29.8% at baseline to 42.9% after two weeks of sucrose rinsing (Figure 1A). Indeed, this finding clearly underlines the well-known role of *Streptococcus* species in carbohydrate metabolism, which is why *Streptococcus* species are considered highly important in the pathogenesis of dental caries [21]. Interestingly, when focusing the analysis solely on the abundance of members of the genus *Streptococcus*, a massive increase in the relative abundance of *Streptococcus salivarius* from 25.8% at baseline to 41.1% was evident (Figure 2C). Importantly, the carbohydrate metabolism of many *Streptococcus* species, including *Streptococcus salivarius*, was thoroughly characterized decades ago using culture-based microbial analysis [22]. Indeed, when used in combination with modern molecular techniques such as transcriptomics and metabolomics, traditional culture-based techniques provide detailed data on genes and pathways involved in carbohydrate metabolism of *Streptococcus* species [23]. However, such analysis most often involves characterization of specific species grown in monoculture in the laboratory, which hampers the possibility to evaluate the phenotypic profile of the species of interest when this is part of a polymicrobial biofilm competing on available nutrients in vivo. Therefore, the use of community-based analysis elegantly demonstrates the blossoming of *Streptococcus salivarius* at the expense of other *Streptococcus* species in conditions with plenty of carbohydrates being available in vivo.

While sugar stress induced immediate and rampant compositional changes in the salivary microbiota, these changes proved to be transient in nature and completely reversible. Accordingly, no differences could be observed when comparing the salivary microbiota at baseline and at week 5 (Figure 1A–C and Figure 2C). Thus, our data show the ability of the salivary microbiota to be able to return to baseline composition in the post perturbation period. Interestingly, this finding is in accordance with data on the recovery profiles of the salivary microbiota after being exposed to antibiotic treatment [24]. Collectively, these findings support the important role of the metabolic resilience of the salivary microbiota [25] in stabilizing homeostasis in the time after the oral cavity has been challenged by short-term stress of various origins.

As opposed to sucrose, xylitol stress had minimal impact on the composition of the salivary microbiota, as no significant alterations were evident by PCA or relative abundance of predominant bacterial genera and species (Figure 3A,C and Figure 4A–E). On the other hand, the combination of xylitol and probiotics had an immediate effect on the salivary microbiota. Specifically, combined use of xylitol and probiotics resulted in compositional changes in the salivary microbiota, clearly visible on PCA (Figure 6A), which were driven by a dramatic decrease in the relative abundance of *Streptococcus* species from 37.6% at baseline to 23.0% after consumption of xylitol and probiotics for two weeks (Figure 5A). Interestingly, when the combined probiotic and xylitol stress was removed after two weeks, the continuous use of probiotics was not sufficient to maintain the changes recorded at week 2, and recurrence to baseline conditions was observed at week 5 (Figure 5A,C and Figure 6B,C). Therefore, the data suggest that the probiotic strains alone were not adequate to impact the salivary microbiota, which is in line with results from a previous study using the same probiotics [26]. On the other hand, the present study shows that xylitol may act as a prebiotic to the strains tested, since an effect on the salivary microbiota was only evident when the two perturbations were simultaneously present. Therefore, the data suggest a potential symbiotic effect of xylitol and probiotic strains on the oral microbiota. Importantly, the combined effect of xylitol and probiotics expressed by a significant decrease in *Streptococcus* species was opposite to the effect of sugar stress. Consequently, it is possible that combined intake of xylitol and probiotics may be protective against the perturbation effect of sugar stress. However, long-term prospective studies with clinical endpoints performed in populations with high levels of carbohydrate intake and high caries prevalence are needed to evaluate whether this strategy could have clinically relevant beneficial effects. 

In the present study, probiotics seemed to have an impact on salivary levels of transferrin. Accordingly, while sugar stress induced a significant increase in salivary transferrin in the sucrose and placebo group, this was not the case in the group that received probiotics during sugar stress (Table 3). Interestingly, in a previous study, the same probiotics did not have an effect on salivary levels of a battery of inflammatory markers including interleukin 1β and tumor necrosis factor α in patients with gingivitis [26]. Multiple explanations may account for these discrepancies, including the nature of the inflammatory molecules studied as well as the different clinical status of the participants in the two studies. Nevertheless, we have previously demonstrated significantly different transferrin levels in saliva in patients with periodontitis and healthy controls [27], which is why these were included in the present study. Future studies are needed to validate if the changes in transferrin levels could be interpreted as an indirect effect of the probiotic strains tested. 

An interesting aspect of the present study was that molecular analysis was based on samples collected by the participants themselves. Indeed, this was only possible since saliva can be collected non-invasively [28]. Furthermore, sampling of saliva requires no other qualifications than what can be disseminated online by short videos. Consequently, sampling could be easily performed with great reproducibility, even though the majority of the participants had no experience with saliva sampling prior to participation. Therefore, conduction of the present study clearly underlines the feasibility of using of saliva-based screening of selected biomarkers in situations where, for one reason or another, it is not practicable to see the person of interest at the dental or the medical doctor’s office [29]. Naturally, this could be the case in many circumstances, including the present, where the study was conducted during a national lockdown caused by the COVID-19 pandemic. Hence, such an approach could also be used when conducting large-scale population surveys or as part of disease monitoring in individuals living in rural areas. 

The present study has several limitations, including the fact that the study design did not include a group, which consumed both xylitol and probiotics during sugar stress. Thus, the data could not be used to answer the question, as to whether combined consumption of xylitol and probiotics counteracts the effect of sugar stress on the salivary microbiota. However, the alterations induced by xylitol and probiotics to the salivary microbiota, as expressed by a significant decrease in *Streptococcus* species, were directly opposite to the sugar-associated modifications of the salivary microbiota. Therefore, data from the present study suggest that combined consumption of xylitol and probiotics may represent an adjunct to traditional caries preventive regimens in individuals with frequent carbohydrate intake. Obviously, future studies are needed to validate this assumption. In addition, oral health status was self-reported, which was a natural consequence of conducting the study during COVID-19 lockdown. However, the majority of participants were dental students of a young age and good general health (Table 1), which is why their self-awareness of oral health status was most likely credible. Furthermore, as a consequence of the COVID-19 lockdown, the patients had to collect biological samples themselves. Therefore, it was not practically possible to collect supragingival plaque samples. Indeed, collection of supragingival plaque samples would have offered the possibility to address the effect of sugar stress on local biofilms involved in initiation of dental caries. However, we have previously demonstrated the composition of the salivary microbiota to reflect compositional changes in local biofilms during coordinated perturbations such as oral hygiene discontinuation and non-surgical periodontal treatment [17,30]. In line with this, the salivary microbiota has been documented as the main donor of bacteria to the supragingival plaque [31]. Therefore, the rampant effect of sugar stress on the salivary microbiota was most likely representative of the supragingival microbiota. Nevertheless, future studies are needed to address this question. Finally, in the present study, we enrolled young individuals with good oral and general health. Indeed, this was a conscious choice, as we wished to use the oral cavity as a model system to characterize the impact of sugar stress in healthy conditions, where the microbiota is not challenged by the presence of oral disease. However, the highly homogeneous study population hampers the generalizability, which is why the data presented could be validated in cohorts with treatment requiring oral disease. 

In conclusion, the data from the present study are the first to reveal the impact of combined consumption of xylitol and probiotics on the salivary microbiota in orally healthy individuals, as expressed by a significant decrease in *Streptococcus* species. Thus, xylitol and the tested probiotic strains may act symbiotically, with augmenting effects on the compositional stability of the salivary microbiota. Future studies are needed to reveal the potential clinical relevance of this finding in caries prevention in individuals with frequent carbohydrate intake. 

## 4. Materials and Methods

### 4.1. Study Design

**Figure 7:**
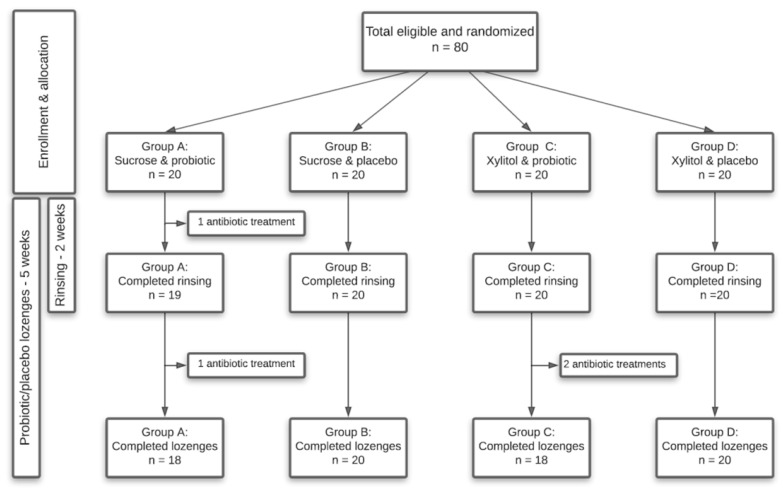
Flowchart of the study.

The present study was a randomized, blinded, four-armed, placebo-controlled clinical trial with a total duration of 5 weeks, carried out from April to May 2020 during the COVID-19 lockdown in Denmark. Randomization was performed at baseline to allocate participants to four different groups, who received either sucrose or xylitol solutions combined with either probiotic or placebo lozenges. Participants were instructed to rinse 6–8 times a day in the initial two weeks of the trial with their assigned solution, while instructions were given to consume the lozenges twice a day throughout the trial period of 5 weeks. Finally, to avoid the risk of infection with COVID-19 as well as comply with the restrictions from the Danish government, participants were instructed to collect saliva samples by themselves at baseline, week 2 and week 5. A timeline of the study is shown in Figure 7.

### 4.2. Study Population

Details of the study population are given in Table 1, which was comprised of 80 self-reported orally and systematically healthy individuals aged 20–32 years, distributed in four groups—A: sucrose and probiotic (*n* = 20); B: sucrose and placebo (*n* = 20); C: xylitol and probiotic (*n* = 20); D: xylitol and placebo (*n* = 20). The sample size was estimated by a power calculation, which was based on data from our recently published study [32]. In order to disclose significant changes in the oral microbiota as a consequence of sugar stress, a power calculation with α = 0.05 and β = 0.2 revealed that 16 subjects were needed in each group. To compensate for drop-outs, we enrolled a total of 20 subjects in each group. The main inclusion criteria for participants were self-reported oral and systemic health and age ≥ 18 years, while exclusion criteria were self-reported treatment requiring dental caries, periodontitis or extensive gingivitis, current smoking, pregnancy, systemic diseases and use of any systemic antibiotics three months prior to study participation. Participants were recruited at the Department of Odontology, University of Copenhagen, and all participants signed informed consent prior to participation. The study was performed in accordance with the Helsinki declaration and approved by the regional ethical committee (H-19086532). Finally, the study was reported to the local data authorization of the Faculty of Health and Medical Sciences, University of Copenhagen (514-0434/19-3000), and registered at ClinicalTrials.gov (UCPH_OI_004) (accessed on 24 March 2021). 

### 4.3. Collection of Samples 

Due to the COVID-19 lockdown, it was decided that participants would conduct the saliva sampling themselves. A total of 4 participants were excluded during the trial due to antibiotic treatment. Therefore, a total of 235 saliva samples were collected at baseline (*n* = 80), week 2 (*n* = 79) and week 5 (*n* = 76). Participants were strictly instructed to conduct the sampling as the first thing in the morning before any food or drink consumption and before their oral hygiene routine. Samples were collected between 6.00 and 9.00 AM, but participants were instructed to individually collect the samples at the same time on the three different sampling days. Participants were briefed through video, verbal and written instructions to collect two different saliva samples. First, a stimulated Salivette^®^ saliva sample was collected as described in our recent study [27], followed by a paraffin-stimulated saliva sample as previously described [33]. Thus, a minimum of 1 mL of stimulated saliva was collected. Participants were instructed to immediately store the samples at −18 °C until pick-up by the examiner (C.L.-O.), whereupon the samples were stored at −80 °C until further analyses. Great effort was made to collect the samples as fast as possible and the majority were collected within 5 h, while all samples were stored at −80 °C within 48 h. 

### 4.4. Sucrose and Xylitol Solution 

Both solutions were made at the University of Copenhagen, and while the sucrose solution was 10%, the xylitol solution was 5% to secure a taste as similar as possible. The solutions were produced with ion-exchanged water, which was sterilized at 121 °C and cooled to room temperature. Likewise, the equipment used for the production together with the dispensed glass bottles for final storage were sterilized to minimize the risk of bacterial growth. In total, 120 L was prepared, allocated as 60 L of 10% sucrose solution and 60 L of the xylitol solution. The sucrose solution was prepared using 6 kg of commercial sugar and prepared in batches of 3 L using a ratio of 100 g of sugar to 1 L of distilled water. The sugar was dissolved using a magnetic stirrer, after which the solution was carefully distributed into 0.5 L sterilized glass bottles and kept refrigerated until dispensed. In the exact same way, 60 L of the 5% xylitol solution was prepared, but using a ratio of 50 g of xylitol to 1 L of distilled water. After distribution, participants were instructed to store the cans in the fridge to avoid bacterial growth. Participants were instructed through video, written and verbal guides to rinse and spread 10 mL of their allocated solution for 30 s 7–8 times a day and with at least 1-h intervals. Furthermore, participants were instructed to avoid any food or drink consumption in the subsequent 15 min to avoid oral clearance and ensure the longest possible exposure. The remaining rinsing fluid was measured at the end of the rinsing period to control compliance. 

### 4.5. Probiotics and Placebo 

The probiotic lozenges contained an equal mix of *Lacticaseibacillus rhamnosus* (formerly *Lactobacillus*) PB01 DSM14870 and *Latilactobacillus curvatus* (formerly *Lactobacillus*) EB10 DSM32307 at a concentration of 1 × 10^9^ CFU/tablet and were the same as used in our recent study [26]. The probiotic strains were originally selected on the basis of in vitro assays, where the chosen strains showed the best immunologic and growth-inhibiting effects on selected oral bacterial species. The lozenges were identical in size, taste and composition with the exception of the probiotic strains, and probiotic as well as placebo lozenges were packed and handed out to the participants in identical pots. Participants were blinded throughout the trial period. Participants were instructed to soak and distribute one lozenge in the oral cavity twice a day, morning and evening, immediately after their oral hygiene routine to ensure presence during the initial biofilm formation. Furthermore, the participants were instructed to avoid any food or drink consumption in the subsequent 30 min to minimize the influence of oral clearance and achieve the longest and, thus, best possible effect. Finally, to supervise compliance, remaining lozenges were collected at the end of the trial. Both probiotic and placebo lozenges were manufactured and provided by Deerland Probiotics and Enzymes A/S, Hundested, Denmark. 

### 4.6. DNA Extraction 

DNA was purified from untreated saliva samples using the ZymoBIOMICS 96 DNA kit, according to the manufacturer’s instructions (Cat.#D4309, Zymo Research, Irvine, CA, USA). Briefly, samples were thawed and diluted to 1:4 in DNA/RNA Shield (Cat.#R1100-250, Zymo Research, Irvine, CA, USA) in the included ZR BashingBead Lysis Tubes. The ZR BashingBead Lysis Tubes were subsequently vortexed at full speed for 40 min in a Horizontal-(24) Microtube Holder mounted on a Vortex Genie 2 (Scientific Industries, Bohemia, NY, USA). After lysis, all samples were frozen at −20 °C until DNA extraction following the manufacturer’s protocol, with optional purification using the included Silicon-A-HRC plate.

### 4.7. Library Preparation

Bacteria 16S V1-3 rRNA gene sequencing libraries were prepared using a custom protocol based on Caporaso et al. (2012) [34]. Up to 10 ng of extracted DNA was used as a template for PCR amplification of the bacterial 16S V1-V3 rRNA gene amplicons. Each PCR reaction (25 μL) contained (12.5 μL) PCRBIO Ultra mix (PCR Biosystems, London, United Kingdom) and 400 nM of each forward- and reverse-tailed primer mix. PCR was conducted with the following program: Initial denaturation at 95 °C for 2 min, 30 cycles of amplification (95 °C for 15 s, 55 °C for 15 s, 72 °C for 50 s) and a final elongation at 72 °C for 5 min. Duplicate PCR reactions were performed for each sample and the duplicates were pooled after PCR. The adaptors contained 16S V1-V3 specific primers: [27F] AGAGTTTGATCCTGGCTCAG and [534R] ATTACCGCGGCTGCTGG. The resulting amplicon libraries were purified using the standard protocol for Agencourt Ampure XP Beads (Beckman Coulter, San Diego, CA, USA) with a bead-to-sample ratio of 4:5. DNA was eluted in 25 μL of nuclease-free water (Qiagen, Hilden, Germany). DNA concentration was measured using a Qubit dsDNA HS Assay kit (Thermo Fisher Scientific, Waltham, MA, USA). Gel electrophoresis using TapeStation 2200 and D1000/High-sensitivity D1000 screen tapes (Agilent, Santa Clara, CA, USA) was used to validate the product size and purity of a subset of sequencing libraries.

### 4.8. DNA Sequencing

The purified sequencing libraries were pooled in equimolar concentrations and diluted to 2 nM. The samples were paired-end sequenced (2 × 300 bp) on a MiSeq system (Illumina, San Diego, CA, USA) using a MiSeq Reagent kit v3 (Illumina, San Diego, CA, USA) following the standard guidelines for preparing and loading samples on the MiSeq system and sequencing approximately 100,000 reads/sample. Then, the >10% PhiX control library was spiked in to overcome low complexity issues often observed with amplicon samples.

### 4.9. Bioinformatic Processing

The base called and demultiplexed Illumina reads were processed using a usearch11 pipeline [35] using forward reads only, matching against the 16S rRNA Human Oral Microbiome Database (HOMD) v. 15.2 [36]. The entire workflow can be summarized in the following steps, all with default settings unless otherwise noted: 1. PhiX spike-in sequences were first filtered from each sample using the usearch11 filter_phix command; 2. The sequences were then filtered based on Q scores using the usearch11 fastq_filter command [37], with max expected errors set to 1.0 (fastq_maxee 1.0) and truncated to 250bp (fastq_trunclen) and, afterwards, concatenated into a single fastq file; 3. The file with all quality checked reads was then dereplicated by using the usearch11 fastx_uniques command, and afterwards, zero radius operational taxonomic units (zOTUs), also known as amplicon sequence variants (ASVs), were generated using the unoise3 command; 4. The taxonomy of the ASVs was then predicted using the SINTAX algorithm [38] (usearch11 sintax) with the settings strand both and sintax_cutoff 0.8 using the HOMD; 5. Finally, an abundance table was generated using the usearch11 otutab command by mapping the zOTUs obtained from step 3 to the PhiX filtered reads from step 1. The results were analyzed in R v. 4.0.2 [39] through the Rstudio IDE using the ampvis package v.2.6.6. Linear discriminant analysis effect size (LEfSe) [40] was used to determine the features (organisms) most likely to explain differences between all combinations of classes (interventions and time points). The analysis was carried out with default settings.

### 4.10. Immunological Analysis and NGAL and Transferrin

Analysis of NGAL and transferrin (ng/mL) was performed as previously described [27]. The samples were thawed at room temperature spun down at 2000G for 5 min and transferred to standard 13-mm analysis tubes. NGAL was analyzed on the Siemens Vista 1500 platform (Siemens, Erlangen, Germany) using an NGAL Test assay (cat. nr. ST001RA), the NGAL Calibrator Kit (cat. nr. ST002CA) and the NGAL Control Kit (cat. nr ST003CA), all from Bioporto Diagnostics, Hellerup, Denmark. Samples with an NGAL concentration over 3000 ng/mL were diluted to 1:4, 1:9 or 1:19 in isotonic NaCl and reanalyzed. Transferrin concentration in saliva was measured on the Vista 1500 platform (Siemens, Erlangen, Germany) using the TRF Flex reagent in urine mode, as the transferrin concentration in saliva is low.

### 4.11. Statistics

The salivary microbiota was characterized and compared by relative abundance, bacterial diversity and visualizing of data by principal component analysis (PCA). Data on relative abundance were corrected for multiple dependent associations using the Benjamini–Hochberg correction [41]. Median values of NGAL and transferrin were compared using a non-parametric Friedman test. For this analysis, a *p*-value < 0.05 was considered significant.

## Figures and Tables

**Table 1:** Background data of the study group.

	Sucrose + Placebo (*n* = 20)	Sucrose + Probiotics (*n* = 20)	Xylitol + Probiotics (*n* = 20)	Xylitol + Placebo (*n* = 20)
Gender, female/male	16/4	16/4	14/6	13/7
Age (mean, range) years	24.2 (21–29)	24.3 (20–32)	24.6 (20–32)	23.7 (20–29)
Dental professions *	11/20	16/20	14/20	17/20

* Dental students, dental hygienist students, dentists, dental hygienists, dentist’s assistants.

**Table 2:** Neutrophil gelatinase-associated lipocalin (NGAL) levels (ng/mL) in saliva expressed as median and 95% CI of median.

NGAL				
	Baseline	Week 2	Week 5	*p*-Value
**Sucrose + probiotics**	1422 (1189–2236)	1780 (1271–2638)	1249 (877–1986)	0.13
**Sucrose + placebo**	1837 (1423–2677)	2208 (775–2761)	2462 (1211–4141)	0.5
**Xylitol + probiotics**	1429 (1053–1757)	1458 (815–2367)	1061 (559–1908)	0.31
**Xylitol + placebo**	1209 (825–2112)	1709 (937–2553)	1399 (914–3000)	0.26

**Table 3:** Transferrin levels (ng/mL) in saliva expressed as median and 95% CI of median.

Transferrin				
	Baseline	Week 2	Week 5	*p*-Value
**Sucrose + probiotics**	4.4 (3.0–7.4)	4.8 (3.5–6.1)	3.8 (2.7–7.1)	0.1
**Sucrose + placebo**	4.5 (3.8–7.5)	5.5 (3.0–6.6)	6.8 (3.9–12.7)	0.04
**Xylitol + probiotics**	4.7 (3.4–7.2)	3.8 (2.6–5.3)	3.9 (2.5–5.0)	0.05
**Xylitol + placebo**	3.7 (2.7–6.1)	4.2 (2.9–6.0)	4.2 (2.5–7.5)	0.77

## Data Availability

Raw sequences have been deposited in European Nucleotide Archive (ENA, www.ebi.ac.uk, accessed on 24 March 2021) with the accession number PRJEB43052.
